# Transcribed sex-specific markers on the Y chromosome of the oriental fruit fly, *Bactrocera dorsalis*

**DOI:** 10.1186/s12863-020-00938-z

**Published:** 2020-12-18

**Authors:** Davide Carraretto, Nidchaya Aketarawong, Alessandro Di Cosimo, Mosè Manni, Francesca Scolari, Federica Valerio, Anna R. Malacrida, Ludvik M. Gomulski, Giuliano Gasperi

**Affiliations:** 1 Department of Biology and Biotechnology, University of Pavia, Pavia, Italy; 2 Department of Biotechnology, Faculty of Science, Mahidol University, Bangkok, Thailand; 3 Department of Genetic Medicine and Development, University of Geneva Medical School, and Swiss Institute of Bioinformatics, Geneva, Switzerland

**Keywords:** Representational difference analysis (RDA), Sex-determination, Chromosome evolution, Transposable element, *Gigyf*, Genetic sexing

## Abstract

**Background:**

The Oriental fruit fly, *Bactrocera dorsalis*, is a highly polyphagous invasive species with a high reproductive potential. In many tropical and subtropical parts of the world it ranks as one of the major pests of fruits and vegetables. Due to its economic importance, genetic, cytogenetic, genomic and biotechnological approaches have been applied to understand its biology and to implement the Sterile Insect Technique, currently a part of area-wide control programmes against this fly. Its chromosome complement includes five pairs of autosomes and the sex chromosomes. The X and Y sex chromosomes are heteromorphic and the highly heterochromatic and degenerate Y harbours the male factor *BdMoY*. The characterization of the Y chromosome in this fly apart from elucidating its role as primary sex determination system, it is also of crucial importance to understand its role in male biology. The repetitive nature of the Y chromosome makes it challenging to sequence and characterise.

**Results:**

Using Representational Difference Analysis, fluorescent in situ hybridisation on mitotic chromosomes and in silico genome resources, we show that the *B. dorsalis* Y chromosome harbours transcribed sequences of *gyf,* (*typo-gyf*) a homologue of the *Drosophila melanogaster Gigyf* gene, and of a non-LTR retrotransposon R1. Similar sequences are also transcribed on the X chromosome. Paralogues of the *Gigyf* gene are also present on the Y and X chromosomes of the related species *B. tryoni*. Another identified Y-specific repetitive sequence linked to *BdMoY* appears to be specific to *B. dorsalis*.

**Conclusions:**

Our random scan of the Y chromosome provides a broad picture of its general composition and represents a starting point for further applicative and evolutionary studies. The identified repetitive sequences can provide a useful Y-marking system for molecular karyotyping of single embryos. Having a robust diagnostic marker associated with *BdMoY* will facilitate studies on how *BdMoY* regulates the male sex determination cascade during the embryonic sex-determination window. The Y chromosome, despite its high degeneracy and heterochromatic nature, harbours transcribed sequences of *typo-gyf* that may maintain their important function in post-transcriptional mRNA regulation. That transcribed paralogous copies of *Gigyf* are present also on the X and that this genomic distribution is maintained also in *B. tryoni* raises questions on the evolution of sex chromosomes in *Bactrocera* and other tephritids.

**Supplementary Information:**

The online version contains supplementary material available at 10.1186/s12863-020-00938-z.

## Background

The Oriental fruit fly, *Bactrocera dorsalis*, is a highly polyphagous invasive species with a high reproductive potential [1]. A native of Southeast Asia, it is one of the most economically destructive pests of fruit and vegetables especially in east Asia. Given its phytophagous nature, it dispersed with the diffusion and implementation of agriculture, while globalization allowed it to establish adventive populations in different tropical and subtropical parts of the world [2, 3]. A recent incursion of this fly into Italy is currently creating concern in Europe [4]. Given its economic importance, genetic, cytogenetic, genomic and biotechnology approaches have been applied to understand its biology and to implement the Sterile Insect Technique (SIT) that is currently used in area-wide control programmes against this fly. The chromosome complement has been characterised [5, 6], a draft genome is available [7], germline transformation and genome editing approaches have been employed [8, 9]. Like other tephritids, the karyotype consists of five pairs of autosomes and a pair of heteromorphic sex chromosomes (X and Y), the heterogametic sex being the male [6]. In *Drosophila* the Y chromosome is not strictly necessary for sex-determination [10], although males that lack the Y chromosome are sterile as the chromosome harbours genes necessary for fertility [11]. Unlike *Drosophila*, the Y chromosome is essential in tephritids [12] as it contains a conserved male determining factor, *Maleness-on-the-Y* (*BdMoY*), that encodes a protein that is necessary and sufficient for male development [13]. The characterization of the Y chromosome in tephritids, apart from elucidation of their primary role in sex determination, it is also of crucial importance to understand their role in male biology. Despite the importance of the Y chromosome in tephritid sex determination their repetitive nature makes them difficult to sequence and characterise [14]. Due to the lack of recombination [15], the Y chromosome in Diptera is transmitted in a clonal manner. The result is a progressive genetic degeneration including the accumulation and rapid turnover of repetitive sequences [16–18]. In particular, evolutionary models to explain the degeneration of the Y chromosome are based on interference among selected mutations on a non-recombining chromosome. Theory and computer simulations have shown that the magnitude of selection interference, and hence the rate of degeneration, depends on the number of functional genes present on the Y chromosome [19].

Only genes under intense selective pressure, such as those involved in sex determination and fertility, are likely to survive on the Y chromosome. Other genes may persist as pseudogenes. The presence of several important genes, such as those related to male fertility, on the *Drosophila* Y chromosome appears to have influenced the evolution of the chromosome [20, 21]. The identification of Y-linked sequences in *B. dorsalis*, as in other tephritids, may tell us much about the repetitive nature, the origin and the evolution of Y chromosomes. The identification of Y-specific sequences will permit the sexing of embryos [22], useful for studying the expression of sex determination and differentiation genes during development. From a practical point of view, a marked Y chromosome could also help the development of genetic control mechanisms for insect disease vectors and agricultural pests [23, 24]. Unfortunately, due to the highly repetitive nature of Y chromosomes and the lack of homology between Y-linked sequences even from related species, the identification of Y-linked sequences remains challenging [11]. Different approaches have been applied to identify Y-linked sequences, such as the chromosome quotient (CQ) [25] and the Y chromosome Genome Scan (YGS) [26] methods used in mosquitoes and *Drosophila* and humans, respectively. Here we employed a Representational Difference Analysis (RDA) approach [27] to identify Y-specific sequences of *B. dorsalis*. We have previously used this technique to identify Y-specific sequences in *Bactrocera oleae* [28]. The identified Y-specific sequences were characterised, and their distributions analysed using mitotic chromosome in situ hybridization. We hypothesize how these repetitive sequences accumulated and were maintained on the Y chromosome during its evolutionary history. Our data reinforce the idea that the sex chromosomes of the Tephritidae may have distinct evolutionary origins with respect to those of the Drosophilidae and other Dipteran families.

## Results

### Identification and characterization of male-specific or male-enriched RDA sequences

**Fig. 1:**
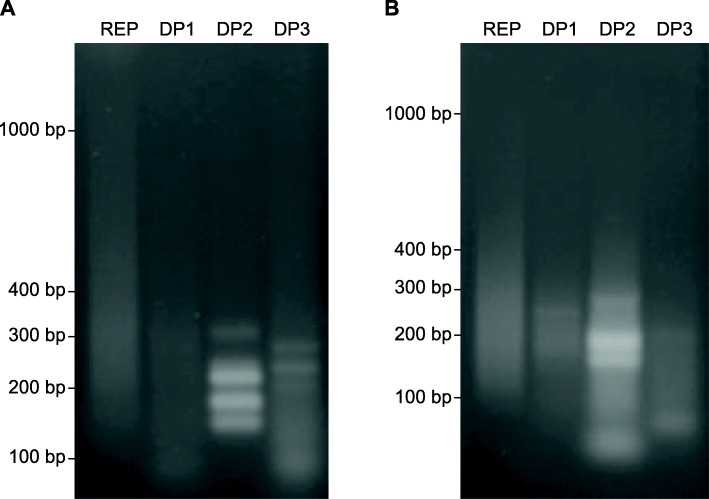
Representational difference products from *Bactrocera dorsalis* genomic DNA digested with *Msp*I (**a**) and *Mse*I (**b**). In each case the Representation (REP) and the three differential products (DP1, DP2 and DP3) are shown

The fragments amplified during each step of the RDA analyses are shown in Fig. 1. Discrete bands were observed in the Difference Product 3 (DP3) of both *Msp*I and *Mse*I RDA libraries. All bands were subsequently isolated by gel electrophoresis, gel eluted, and sequenced. The DP3 of the *Msp*I and *Mse*I libraries produced a total of 19 sequences, consisting of twelve and seven from each library, respectively. After sequence assembly, four consensus sequences (contigs) were identified; three from the *Msp*I library, contig1 (126 bp), contig2 (150 bp), and contig3 (217 bp), and one from the *Mse*I library, contig4 (179 bp).

**Fig. 2:**
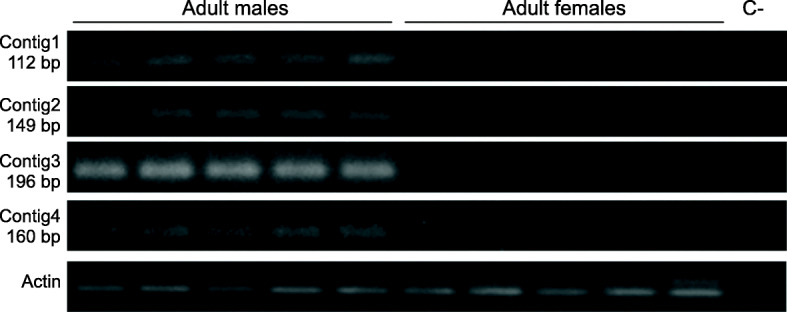
Amplification of fragments of the original contigs 1–4 from genomic DNA derived from adult male and female individuals. Amplification of an actin fragment from both sexes as a control for DNA integrity. The sizes of the amplified fragments are indicated. The negative control (C-) without genomic DNA template is indicated

**Fig. 3:**
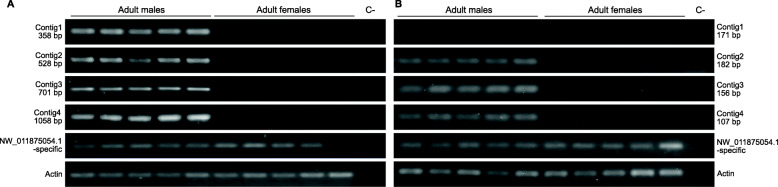
Amplification of fragments of the extended contigs 1–4 and the NW_011875054.1-specific sequence from genomic DNA (**a**) and from cDNA (**b**) derived from adult male and female individuals. Amplification of an actin fragment from both sexes as a control for DNA and cDNA integrity. The sizes of the amplified fragments are indicated. The negative control (c-) without genomic DNA or cDNA template is indicated

Primers based on the sequences of contigs1 to 4 (Additional file 1 - Table S1) were used to amplify male and female genomic DNA from the Saraburi strain. Amplicons were obtained in both sexes, although the male amplicons appeared to be more intense compared to those from the female samples. Increasing the annealing temperature by 8 °C resulted in male-only amplicons (Fig. 2). The contigs were subsequently extended by inverse PCR, to obtain sequences of 770 bp, 553 bp, 738 bp, and 1111 bp, respectively. Primers designed on these extended sequences amplified products only in male samples (Additional file 1 - Table S1, Fig. 3a).

All contigs were subsequently analysed using Blastn against the *B. dorsalis* genome (ASM78921v2) and the non-redundant (nr) nucleotide database (Table 1). The extended contig1 represents a repetitive sequence which consisted of two 385 bp units sharing 98% identity (Additional file 2 - Figure S1). It did not share significant similarity with any sequence in the nr database or with the *B. dorsalis* genome sequence (Table 1).

**Table 1:** Similarities of the contigs with sequences within the *Bactrocera dorsalis* genome (ASM78921v2), non-redundant nucleotide and protein (nr) databases using Blastn and Blastx

Contig	Length (bp)	Database	Blastn best hits	Accession no.	e value	% Identity/Similarity^a^
contig1	770	*B. dorsalis* genome	no significant hit	–	–	–
		nr	no significant hit	–	–	–
		nr (blastx)	no significant hit	–	–	–
contig2	553	*B. dorsalis* genome	Unplaced genomic scaffold03090	NW_011873311.1	0.0	99
			Unplaced genome scaffold01347	NW_011875054.1	7e-80	81
		nr	*Bactrocera dorsalis* GIGYF family protein CG11148 (LOC109580051), partial mRNA	XM_019992828.1	0.0	99
		nr (blastx)	*Bactrocera dorsalis* GIGYF family protein CG11148, partial	XP_019848387.1	8e-74	99/99
contig3	738	*B. dorsalis* genome	Unplaced genomic scaffold00825	NW_011875576.1	2e-112	86
			Unplaced genome scaffold01347	NW_011875054.1	3e-104	80
		nr	*Bactrocera latifrons* PERQ amino acid rich with GYF domain-containing protein CG11148 (LOC108976173)	XM_018945108.1	1e-111	82
		nr (blastx)	*Bactrocera tryoni* GYF-like protein	QCX41583.1	4e-28	91/92
contig4	1111	*B. dorsalis* genome	*Bactrocera dorsalis* strain Punador unplaced genomic scaffold04668	NW_011871734.1	0.0	83
			*Bactrocera dorsalis* strain Punador unplaced genomic scaffold01946	NW_011874455.1	3e-95	78
		nr	*Bactrocera tryoni* non-LTR retrotransposon R1	KU543678.1	4e-113	82
		nr (blastx)	*Bactrocera tryoni* hypothetical protein	AMS38360.1	2e-51	77/82

^a^Similarity for blastx searches

**Fig. 4:**
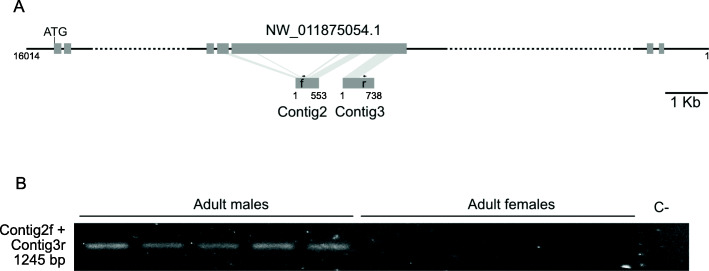
Representation of the localisation of regions of similarity between scaffold NW_011875054.1 and contigs 2 and 3 (**a**), and amplification product using primers within contig2 and contig3 that confirm that they are fragments of the same or similar copies of a *gyf*-like gene (**b**). Similarity between the sequences is indicated by shading. Grey rectangles represent putative exons; dotted lines represent unsequenced padded regions in scaffold NW_011875054.1 (**a**). The negative control (C-) without genomic DNA template is indicated (**b**)

Extended contig2 shares 99% identity with part of scaffold03090 (NW_011873311.1) of the *B. dorsalis* genome which represents a homologue of the PERQ amino acid-rich with GYF domain-containing protein CG11148, GIGYF (XM_019992828.1) (Table 1). Contig2 contains fragments of two exons interrupted by a short intron of 58 bp (Additional file 3 - Figure S2). Extended contig3 shares 86% identity with *B. dorsalis* genome scaffold00825 (NW_011875576.1) and 82% identity with a *Bactrocera latifrons* CG11148-like GIGYF domain-containing protein gene (XM_018945108.1) (Table 1). Contig 3 contains a 268 bp ORF (Additional file 4 - Figure S3). Both contigs 2 and 3 also share hits with parts of an additional *B. dorsalis* genome scaffold (scaffold01347, NW_011875054.1) with 81 and 80% identity, respectively (Fig. 4a). To confirm that the contigs 2 and 3 were adjacent fragments of the same, or similar copies, of an CG11148-like GIGYF gene, contig2f and contig3r primers were used for genomic DNA amplification. An amplification product of 1245 bp was obtained (Fig. 4b). Cloning and sequencing of this product (Additional file 5 - Figure S4) confirmed that contigs 2 and 3 are adjacent sequences of one or more copies of the CG11148-like GIGYF gene. The sequence shares 99% identity with scaffold03090 (NW_011873311.1), that with the highest similarity to contig2, and 82% identity with scaffold01347 (NW_011875054.1).

**Fig. 5:**
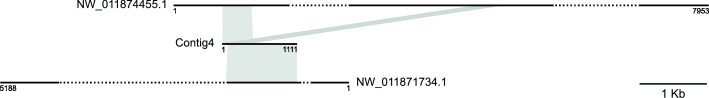
Representation of the localisation of regions of similarity between contig4 and scaffolds NW_011874455.1 and NW_011871734.1. Similarity between the sequences is indicated by shading. Dotted lines represent unsequenced padded regions in the scaffolds

The sequence of extended contig4 (Additional file 6 - Figure S5) straddled two genome scaffolds sharing identity with scaffold04668 (NW_011871734.1) and scaffold01946 (NW_011874455.1). Scaffold04668 shared 83% identity with bases 87–1111 of contig4 whereas two parts of scaffold01946 separated by 3.3 kb shared 78 and 77% identity with bases 6–433 of contig4 (Fig. 5). This region of contig4 also shared significant identity (> 72%) with at least another ten scaffolds, suggesting that this sequence is repetitive in the genome. The contig4 sequence shares identity with a sequence annotated as a *Bactrocera tryoni* non-LTR retrotransposon (Table 1).

### Transcriptional profiles of male-specific and male-enriched sequences in adults of *B. dorsalis*

Contig1 was not transcribed in adults of both sexes (Fig. 3b). Contigs 2 and 3 that showed high similarity to *B. dorsalis* genome scaffolds NW_011873311.1 (99%) and NW_011875576.1 (86%) were transcribed only in male individuals (Fig. 3b). The secondary similarity of contigs 2 and 3 with scaffold NW_011875054.1 (see previous section) suggested that additional *gyf*-like gene sequences (NW_011875054.1) were present in the genome. This stimulated the design of NW_011875054.1-specific primers that would amplify only a fragment from that *gyf*-like gene and not from those corresponding to contigs 2 and 3 (i.e. NW_011873311.1 and NW_011875576.1) (Additional file 7 - Figure S6). These NW_011875054.1-specific primers amplified fragments from both sexes using both genomic DNA or cDNA derived from adult males or females (Fig. 3a and b) suggesting that this *gyf* sequence may be present on either an autosome or the X chromosome. Contig4, that shared identity with scaffolds NW_011871734.1 and NW_011874455.1, was transcribed only in males (Fig. 3b).

### Chromosomal locations of the isolated contig sequences

**Fig. 6:**
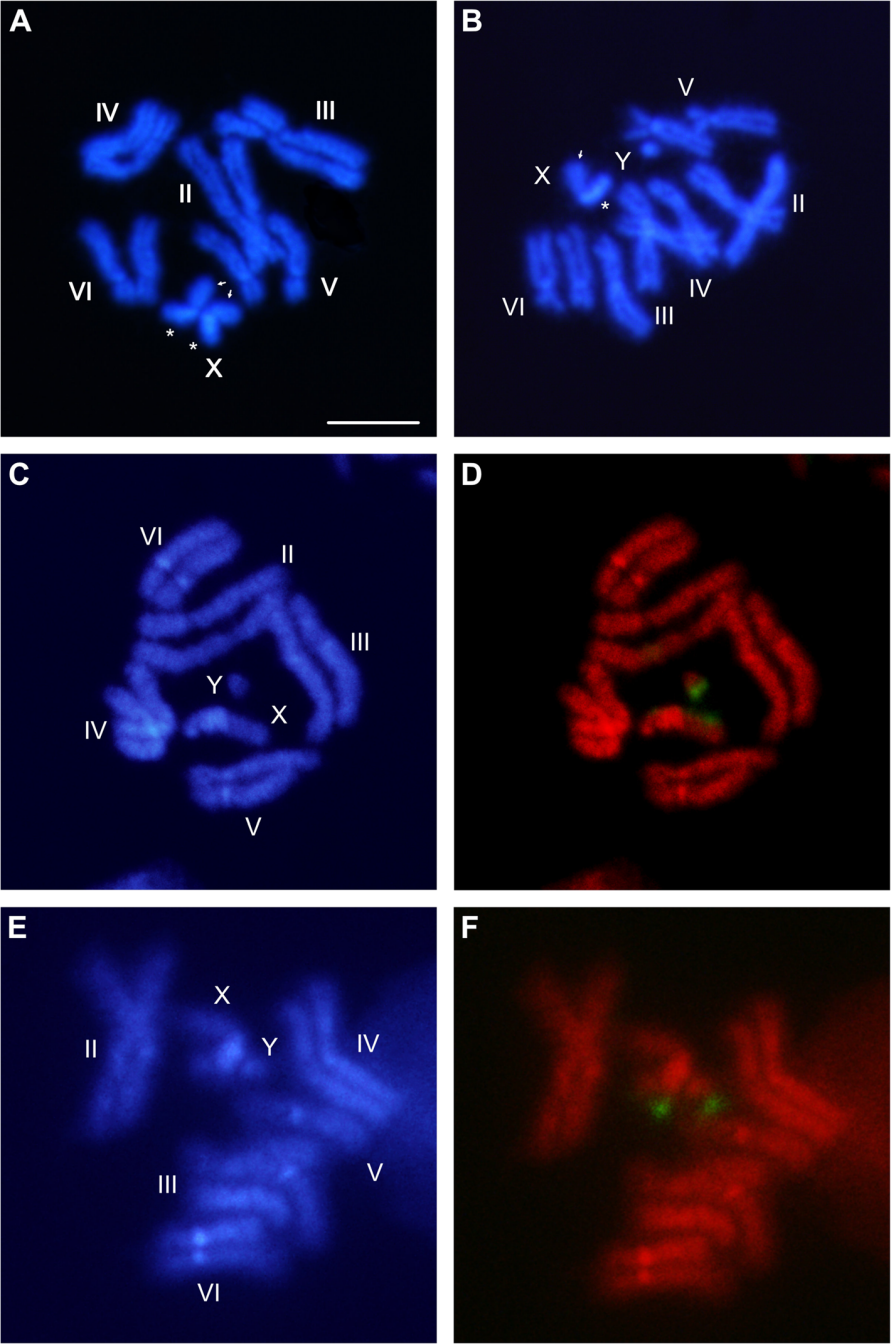
Mitotic chromosomes of *Bactrocera dorsalis*. DAPI stained mitotic karyotypes of a female (**a**) and male (**b**) individual. DAPI stained male karyotypes (**c**, **e**) and respective FISH of contig3 (**d**) and contig4 (**f**) probes. Scale bar represents 5 μm

The DAPI stained mitotic chromosome sets of males and females were used for FISH analysis in order to verify the chromosomal locations of the contig3 and contig4 sequences. DAPI staining permitted the allocation of these sequences also to the hetero/euchromatin level, i.e. in DAPI+ and DAPI- regions, respectively (Fig. 6a and b). Apart from the brightly staining centromeres, the autosomes do not show consistently bright fluorescent bands, while the X and Y chromosomes display characteristic banding patterns. One X arm is marked with a large, bright, DAPI+ fluorescent heterochromatic band encompassing a great part of its length (Fig. 6a and b, asterisk). The opposite arm stains less intensely and shares characteristics of both euchromatin and heterochromatin (Fig. 6a and b, arrow). On the Y chromosome, a DAPI+ area covers almost the entire chromosomal length, apart from regions at the tip of the long arm. FISH on mitotic chromosomes from male and female larvae, using the contig3 fragment (738 bp, Table 1) as a probe, permitted the localization of this sequence to two different positions: one on the euchromatic tip of the long arm of the Y chromosome, and another on the euchromatic arm of the X chromosome (Fig. 6c and d, respectively). Similarly, the extended contig4 produced two hybridization signals: one again at the euchromatic tip of the long arm of the Y chromosome and the other on the euchromatic tip of the short arm of the X chromosome (Fig. 6e and f). These hybridization data confirm the presence of *gyf*-like and of non-LTR retrotransposon-related sequences on both the X and Y sex chromosomes of *B. dorsalis*.

### Contig1 is linked to *BdMoY*, providing a marker for sexing embryos

**Fig. 7:**
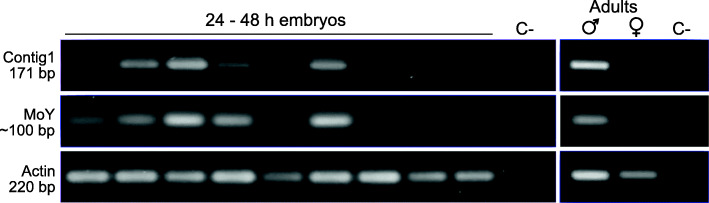
Amplification of the contig 1 and *Maleness-on-the-Y* (*MoY*) fragments from genomic DNA derived from single 24–48 h embryos and from an adult male and female. Amplification of an actin fragment from embryos and adults as a control for DNA integrity. The sizes of the amplified fragments are indicated. The negative control (C-) without genomic DNA template is indicated

Given the repetitive nature and specificity of contig1 for the *B. dorsalis* Y chromosome, the contig1 primers were used to amplify genomic DNA derived from single 24–48 h embryos (Fig. 7). The same single embryo DNAs were used to amplify *BdMoY*, the orthologue of *MoY*, the *Ceratitis capitata* sex determining factor, located on the Y chromosome [13]. The housekeeping gene, *actin*, gave amplification products in all the embryo DNA samples, indicating the presence and integrity of the DNA. In each embryo, as in adult flies, the amplification products using the contig1 and *BdMoY* primers are concordant. This indicates the linkage of contig1 and *BdMoY* on the Y chromosome. The contig1 primers thus provide an unambiguous marker for sexing individuals also at the embryonic stage for this pest species.

## Discussion

Using Representational Difference Analysis (RDA), fluorescent in situ hybridisation on mitotic chromosomes and in silico sequence analyses we provide evidence that the *B. dorsalis* dot-like Y chromosome harbours, in addition to repetitive sequences, transcribed sequences of a homologue of the PERQ amino acid-rich with GYF domain-containing protein CG11148 gene (*typo-gyf)* and non-LTR retrotransposon-like sequences. Intriguingly, similar sequences are also present on the X chromosome. These findings open interesting avenues of investigation both at the evolutionary and applicative levels.

### The Y chromosome harbours male-specific repetitive sequences and a transposable element

The RDA enrichment of Y chromosome sequences revealed the presence of a Y-specific repetitive sequence (contig1) consisting of two short units, and of a transcribed non-LTR retrotransposon R1 (contig4). Based on the highly heterochromatic and degenerate nature of the *B. dorsalis* Y chromosome [6], the enrichment of repetitive sequences and transposable elements (TEs) is expected. Enrichment of TEs on the highly heterochromatic Y chromosomes of other tephritids has been demonstrated [28–31]. Of particular interest is the presence of the non-LTR retrotransposon R1, which is also present on the X chromosome. It is known that non-LTR retrotransposons R1 and R2 have persisted in ribosomal RNA gene loci (rDNA) since the origin of arthropods despite their continued elimination by the recombinational mechanisms of concerted evolution [32]. No information are available on the chromosomal location of rDNA in *B. dorsalis*, but the association of genes encoding ribosomal RNA with sex chromosomes has been demonstrated for *B. oleae* [33] and it seems to be a general feature for tephritids [12, 29, 34, 35] and for some other dipteran groups. This association of rRNA genes with the sex chromosomes may be due to the highly heterochromatic nature of these chromosomes in the Diptera, as rRNA genes are often found in heterochromatic regions [33, 36, 37]. On this basis we can suppose that the presence of non-LTR retrotransposon R1 on the Y and X chromosomes of *B. dorsalis* might be associated to the possible presence of the rRNA gene loci on these sex chromosomes.

### The Y-specific repetitive sequences are useful for embryo molecular karyotyping

We have identified sequences that represent markers for the Y chromosome and that can be utilised for sexing individuals, or fragments of individuals at any developmental stage, including embryos. Of particular interest is contig1, a repetitive sequence that is specific to the Y chromosome, in linkage with *BdMoY*, an orthologue of the *C. capitata MoY* male-determining factor [13]. Meccariello and colleagues [13] demonstrated that reduced expression of *BdMoY* in *B. dorsalis* feminised adults emerging from XY karyotype embryos, proving the conservation of its functional role in determining the male fate also in this species. Having a robust diagnostic marker associated with *BdMoY* will facilitate studies on how *BdMoY* regulates the male sex determination cascade during the embryonic sex-determination window. Moreover, the availability of robust embryonic sexing permits tracking the onset of embryonic development in terms of the timing of cellular blastoderm formation, as previously shown in *C. capitata* [22]. Y-specific markers are also useful for tracing the inheritance of the chromosome in different crosses and in monitoring the stability of Y-translocated strains. The contig1 repetitive sequences are specific to *B. dorsalis*, although this apparent specificity may be an artefact due to the lack of complete genomes available for the *Bactrocera* species. Should the contig1 sequences be specific to this species, it would facilitate the development of robust molecular diagnostic markers to discriminate species (i.e. DNA bar-coding) and sexes within a species.

### The Y chromosome harbours transcribed gene sequences

*Bactrocera dorsalis* provides an additional example that tephritid Y chromosomes, despite their high degeneracy and heterochromatic nature, harbour transcribed sequences, in addition to the *MoY* factor. This had previously been shown for the Y chromosomes of *B. oleae* and *B. tryoni* [28, 38]. These Y-linked sequences require further investigation at the functional level.

Indeed, in *B. oleae*, a large inter-chromosomal duplication containing a transcribed fragment of an *importin-4*-like gene was identified on the Y chromosome. A similar *importin-4* gene fragment is also present on the X chromosome [28]. Importin genes code for nuclear import receptors, that import arginine-serine-rich (SR) proteins into the nucleus as they recognize the SR domains as nuclear localisation signals [39, 40]. Interestingly, a key gene in the sex-determination cascade, *Bo-transformer* [41], is a member of the SR protein superfamily and is directly or indirectly regulated by the *BoMoY* male-determining factor located on the Y chromosome [13, 22]. The presence of *importin-4* on the Y chromosome is probably limited to *B. oleae*, as attempts to isolate related sequences from the Y chromosomes of four other *Bactrocer*a species, including *B. dorsalis*, were unsuccessful [28].

The *B. dorsalis* Y chromosome also contains fragments of genes that may have retained important functional roles. It harbours transcribed copies of a *typo-gyf*-like sequence and a similar sequence, *gyf*, is present also on the X chromosome. Indeed, contigs 2 and 3 represent paralogues of the *Drosophila Gigyf* (*gyf*) gene (FBgn0039936) which has an important role in post-transcriptional mRNA regulation [42]. *Gigyf* is highly expressed in embryonic stages and codes for GIGYF protein that forms a complex with eukaryotic initiation factor 4E homologous protein to elicit translational repression and promotes target mRNA decay. Paralogues of the *gyf* gene were identified in another *Bactrocera* species, *B. tryoni* [38]. About 41 copies, referred to as *typo-gyf*, are present on the *B. tryoni* Y chromosome. Another copy of the gene, *gyf*, is present on the X chromosome. Choo and colleagues [38] suggest that a duplication that gave rise to the Y-linked *typo-gyf* copies occurred in *Bactrocera* between 5.5 and 10.6 MYA, before the split that gave rise to among others, *B. tryoni* and *B. dorsalis*, but after the split that gave rise to *B. oleae* [38]. The extent of copy number expansion in *B. dorsalis* needs to be determined, but at least one X chromosome copy of the *gyf* gene (sequence NW_011875054.1) and at least two Y chromosome *typo-gyf* copies (of which contigs 2 and 3 are fragments) are present. It has been suggested that duplication and subsequent gene conversion may play a role in the evolution and function of Y-linked genes [43]. Whether this is also the case for the *gyf* copies on the *Bactrocera* spp. Y chromosomes is a question that deserves attention. Mahajan and Bachtrog [19] hypothesised that the linkage of genes to the Y chromosome could be the result of their ancestral occurrence on an autosome that subsequently became a sex chromosome. The genes would, of course, have had to escape degeneration. Alternatively, the Y-linked genes could have been secondarily transposed to the Y chromosome. If the first hypothesis were correct, one would expect that the genes’ closest paralogues should be on the X chromosome. If the second hypothesis were true, their closest paralogues should be autosomal [19]. We can thus speculate that the presence of the *typo-gyf* copies on the Y chromosomes and *gyf* on the X chromosomes of *B. dorsalis* and *B. tryoni* may be the consequence of the common origin of the sex chromosomes from an ancestral autosome. *Bactrocera dorsalis* and *B. tryoni* are tightly related species [5]. However, whether the *typo-gyf* fragments identified on the Y chromosome represent genes that have escaped degeneration and are functional remains to be seen. It is noteworthy, however, that the heterochromatin may be a preferential location for ancestral Y genes, as their regulatory machinery will have evolved in a heterochromatic environment on the ancestral Y chromosome [44].

## Conclusions

We have shown that RDA in association with genome, chromosomal and FISH analyses, are a good approach for an initial examination of the Y chromosome of *B. dorsalis.* In the absence of a Y chromosome deep sequencing resource, our approach can provide a general picture of its composition and represents a starting point for deeper applicative analyses and evolutionary considerations. As we have demonstrated, the identification of repetitive sequences can provide a useful Y chromosome marking system linked to the *MoY* male factor. Moreover, we have shown that the Y chromosome harbours transcribed sequences that are conserved on the Y chromosome of a related species, *B. tryoni,* and in both species are also present on the X chromosome. This raises questions on the evolution of the sex chromosomes in *Bactrocera* and tephritid flies in general. Indeed, the tephritid sex chromosomes have a different evolutionary history within Diptera. Within the Tephritidae, the Y chromosomes share some conserved features: they are largely degenerate with much repetitive DNA. According to Vicoso and Bachtrog [45], the dot-like Y chromosome segregated as the sex chromosome in Brachycera lineages that differentiated early in the higher Diptera, suggesting that it is the ancestral sex chromosome of all higher Diptera. This Y chromosome has remained the sex chromosome in several major fly lineages, including the true fruit flies, Tephritidae, and the highly derived calyptrate flies, such as the Calliphoridae and Sarcophagidae. This suggests that this chromosomal element has been sex-linked for over 200 million years of evolution in many higher fly families. The X chromosomes of the Tephritidae and the related Muscidae are not homologous to the *Drosophila* X chromosome [11]. They are completely heterochromatic, carry very few genes, and the genes orthologous to *Drosophila* X-linked genes are autosomal in these species [46]. Thus, in the Tephritidae and Muscidae, the X chromosome probably replaced the former X chromosome.

## Methods

### Flies

A strain of *B. dorsalis*, originally derived from wild flies collected from Saraburi (Thailand), was obtained from the FAO/IAEA Agriculture and Biotechnology Laboratory (Seiberdorf, Vienna, Austria) with the permission of the Italian Ministry of Health, Directorate General for Animal Health and Veterinary Medicines, Rome, Italy. The adults and larval stages were maintained at 24–26 °C with 60–65% relative humidity and a 12:12 h (light: dark) photoperiod. The strains were reared using standard methods [47].

### Genomic DNA preparations

In order to remove superficial contaminants, male and female adult flies were washed in 10% SDS, rinsed with milliQ (Millipore) water, washed with 50% Clorox, rinsed with milliQ water, washed in 70% ethanol and finally rinsed in sterile water, for 3 min each wash step. Genomic DNA was extracted from the legs of each fly using the method reported in Baruffi and colleagues [48]. Following treatment with RNase A, the DNA was extracted with phenol-chloroform, precipitated with ethanol and resuspended in TE buffer (10 mM Tris-HCl, pH 8, 1 mM EDTA). Qualification and quantification of genomic DNA was performed using a Nanodrop ND-1000 spectrophotometer (Nanodrop Technologies Inc., Wilmington, DE, USA).

### Representational Difference Analysis (RDA)

Based on the positive results that we obtained using the Representational Difference Analysis (RDA) method on the Y chromosome of *Bactrocera oleae* [28], we applied the same method as an initial scan of *B. dorsalis* to identify Y-specific or Y-enriched sequences. Briefly, pools of genomic DNA were separately extracted from legs of 60 male and female adults from the Saraburi strain. The genomic DNA was then digested with two four-cutter endonucleases: a CG-rich region cutter (*Msp*I) and an AT-rich region cutter (*Mse*I). The R, J and N series of adaptors were those used by Gabrieli and colleagues [28] (Additional file 8 - Table S2). Male and female Representations were initially generated by PCR of R-adaptor-ligated genomic fragments. The optimum amounts of initial DNA input were 0.2 ng and 8 pg for the *Msp*I- and *Mse*I male and female Representations, respectively. Adaptors were subsequently removed from the Representations by digestion and the products were purified by spin-column purification kit (PureLink PCR micro Kit, Invitrogen). To generate the tester, the J adaptors were ligated to the male Representations, while the female Representations were used as driver. In the first subtractive hybridization, the ratio of male-derived tester and female-derived driver was 1:100. Following the subtractive hybridization, the male-specific DNA was amplified using the J adaptors as primers, to generate the Differential Product 1 (DP1). The DP2 was obtained using a new DNA tester generated by replacement of the DP1 J adaptors for N adaptors. This second round used 1:800 of tester and female-derived driver ratio. Likewise, the DP3 was obtained using a DNA tester generated by replacement of the DP2 N adapters for J adaptors and the ratio of tester/driver was 1:40000.

### Cloning and sequencing of differential product 3 (DP3) sequences

The male-specific bands present in the DP3 were gel eluted and cloned using the TOPO TA cloning kit (Invitrogen). Positive clones were selected, and the inserted size was quantified by *Eco*RI digestion and gel electrophoresis. Clone inserts were sequenced on both strands (Macrogen Europe).

### PCR amplifications of DP3 sequences from male and female genomic DNA

Male and female genomic DNA from five *B. dorsalis* individuals of the Saraburi strain were amplified using primers designed on the DP3 sequences (Additional file 1 - Table S1). PCRs were performed in 15 μl volume containing ~ 10 ng *B. dorsalis* male or female genomic DNA, 1x Buffer, 1.5 mM MgCl_2_, 25 mM dNTPs, 10 μM of each primer and 1 unit *Taq* DNA polymerase (Invitrogen). Amplifications were performed using a Mastercycler Nexus Gradient (Eppendorf) using the following conditions: 94 °C for 4 min, 30 cycles of 94 °C for 30 s, T_a_°C (Additional file 1 - Table S1) for 30 s and 72 °C for 30–60 s, and a final extension at 72 °C for 10 min. Amplification products were electrophoresed on 1.5% agarose gels and visualized by exposure to UV light after ethidium bromide staining.

### Inverse PCR

Approximately 1.5 μg genomic DNA was completely digested using different restriction enzymes (i.e. *Cfo*I, *Taq*I, *Mse*I, *Msp*I, and *Mae*I). Digested DNA was precipitated, self-ligated in 500 μl at 16 °C for 24 h using T4 DNA ligase (Invitrogen), and subsequently precipitated. The pellet was dissolved in 150 μl 10 mM Tris pH 8.5.

Inverse PCRs were performed in 50 μl volume containing 5 μl self-ligated DNA, 1x buffer, 3.5 mM MgCl_2_, 2 mM dNTPs, 10 μM of each primer and 5 units *Taq* polymerase. Amplifications were performed using a Mastercycler Nexus Gradient using the following conditions: 95 °C for 1 min, 6 cycles of 94 °C for 30 s, *T*_a_ for 45 s and 72 °C for 5 min, every cycle the *T*_a_ was lowered by 1.5 °C, followed by 25 cycles of 94 °C for 30 s, *T*_a_-7.5 °C for 45 s and 72 °C for 5 min and a final extension at 72 °C for 6 min. To verify inverse PCR products, nested PCRs were carried out as described above following PCR conditions: 94 °C for 5 min, 29 cycles of 94 °C for 30 s, *T*_a_ (based on the primer pair) for 1 min, 72 °C for 2 min 72 °C and elongation at 72 °C for 5 min.

Inverse PCR/nested PCR products were gel eluted and cloned using the TOPO TA cloning kit and sequenced on both strands.

### Sequence analyses

Sequences of all contigs were analysed using BLAST family of programs from the National Centre for Biotechnology Information (NCBI, USA) [49]. In addition, the sequences were also characterized using BLAST against the *B. dorsalis* strain Punador genome assembly [50]. Schematic genome localization was therefore performed.

### Transcription of contig sequences in adults of *B. dorsalis*

Total RNA was extracted from 10-day old adults using TRIzol™ reagent (Invitrogen) according to manufacturer’s protocol. Further purification was performed using DNA-*free* DNA removal kit (Ambion). Synthesis of cDNA was performed using 200 ng RNA in 20 μl volume using the iScript™ cDNA Synthesis kit (BioRad). Transcriptional profiles were assessed by RT-PCR using primers listed in Additional file 8 - Table S2. The products were analysed on 1.5% agarose gel electrophoresis.

### Chromosome preparation and fluorescence in situ hybridization (FISH)

Mitotic chromosome spreads were obtained from the brains of fourth-instar larvae. The brains were isolated in PBS pH 7.5, transferred to cold hypotonic solution (1% sodium citrate) and incubated for 10 min at room temperature. The brains were then transferred to methanol-acetic acid 3:1 solution for 4 min at room temperature. Subsequently, 100 μl 60% acetic acid was added to the material for chromosome fixation and the brains were macerated and transferred to a pre-heated (65 °C) microscope slide for drying. The slides were stained with DAPI (4′,6-Diamidine-2′-phenylindole dihydrochloride; 10 ng/ml in 4xSSC) which produces a pattern similar to Hoechst 33258, which in *Drosophila* stains heterochromatic regions [51]. Fluorescence in situ hybridizations using contigs 3 and 4 as probes were performed on mitotic chromosome preparations obtained from the larvae. The probes were labelled using the Biotin High Prime kit (Roche) and detection of hybridization signals was performed using the Alexa Fluor 594 Tyramide Signal Amplification Kit (Invitrogen). Chromosomes were counterstained and mounted using the VECTASHIELD mounting medium (Vector Laboratories, Burlingame, CA, USA). Hybridization and DAPI fluorescence signals were visualized through appropriate filters using a Zeiss Axioplan microscope. Images were captured using an Olympus DP70 digital camera with exposure times of 0.5 and 0.2 s for rhodamine and DAPI, respectively.

### Amplification of contig sequences from DNA of single embryos

Eggs were collected over a 24 h period. The eggs were maintained at 24–26 °C for an additional 24 h before dechorionation (using 1.5–2% hypochlorite solution), the eggs were then repeatedly washed in distilled water and individually transferred to 1.5 ml microcentrifuge tubes. DNA was extracted as previously described. The DNA from individual embryos was amplified using the Actin, *MoY* and contig1-171f/contig1-171r primer sets (Additional file 1 – Table S1).

## Supplementary Information

**Additional file 1:** **Table S1.** Primer pairs based on the 4 contigs.

**Additional file 2:** **Figure S1.** Sequence of the extended contig1 showing the positions of the primers. The grey box indicates the extent of the first repetition unit.

**Additional file 3:** **Figure S2.** Sequence of the extended contig2 showing the positions exons (yellow rectangles) and primers.

**Additional file 4:** **Figure S3.** Sequence of the extended contig3 showing the positions of an exon (yellow rectangle) and primers.

**Additional file 5:** **Figure S4.** Sequence spanning contigs 2 and 3 (including the complete contig 2 and 3 sequences).

**Additional file 6.Figure S5**. Sequence of the extended contig4 showing the positions of a putative exon (yellow rectangle) and primers.

**Additional file 7:** **Figure S6.** Alignment of part of the extended contig2 and Scaffold01347 (NW_011875054.1), indicating the positions of primers designed to amplify preferentially the NW_011875054.1 sequence.

**Additional file 8:** **Table S2.** Primers for Representational Difference Analysis (RDA). The R- primers were used in the preparation of the initial amplicon representations, and the J- and N- primers were used for odd and even hybridization-amplifications, respectively.

**Additional file 9.** Unedited gel images from Figs. 1, 2, 3, 4 and 7.

## Data Availability

The sequence data generated during this study are available in GenBank (accession numbers MT978089 - MT978097).

## Abbreviations

BLAST: Basic Local Alignment Search Tool
bp: base pair
cDNA: complementary DNA
CQ: Chromosome quotient
DAPI: 4′,6-Diamidine-2′-phenylindole dihydrochloride
DNA: Deoxyribonucleic acid
DP: Difference Product
FISH: Fluorescence In Situ Hybridization
GYF: Glycine-tyrosine-phenylalanine
kb: kilobase
LTR: Long Terminal Repeat
mRNA: Messenger ribonucleic acid
NCBI: National Center for Biotechnology Information
ORF: Open reading frame
PBS: Phosphate Buffered Saline
PCR: Polymerase chain reaction
RDA: Representational Difference Analysis
rDNA: ribosomal DNA
REP: Representation
RNA: Ribonucleic acid
rRNA: ribosomal RNA
SIT: Sterile Insect Technique
SR proteins: arginine-serine-rich proteins
SSC: Saline Sodium Citrate buffer
YGS: Y chromosome Genome Scan

## References

[1] CABI Invasive Species Compendium: *Bactrocera dorsalis* (Oriental fruit fly) [https://www.cabi.org/isc/datasheet/17685]. Accessed 12 February 2020.

[2] Aketarawong N, Guglielmino CR, Karam N, Falchetto M, Manni M, Scolari F, Gomulski LM, Gasperi G, Malacrida AR (2014). The oriental fruitfly *Bactrocera dorsalis s.s*. in East Asia: disentangling the different forces promoting the invasion and shaping the genetic make-up of populations. Genetica.

[3] Khamis FM, Karam N, Ekesi S, DE Meyer M, Bonomi A, Gomulski LM, Scolari F, Gabrieli P, Siciliano P, Masiga D (2009). Uncovering the tracks of a recent and rapid invasion: the case of the fruit fly pest *Bactrocera invadens* (Diptera: Tephritidae) in Africa. Mol Ecol.

[4] Nugnes F, Russo E, Viggiani G, Bernardo U (2018). First record of an invasive fruit fly belonging to *Bactrocera dorsalis* complex (Diptera: Tephritidae) in Europe. Insects.

[5] Augustinos AA, Drosopoulou E, Gariou-Papalexiou A, Bourtzis K, Mavragani-Tsipidou P, Zacharopoulou A (2014). The *Bactrocera dorsalis* species complex: comparative cytogenetic analysis in support of Sterile Insect Technique applications. BMC Genet.

[6] Zacharopoulou A, Augustinos AA, Sayed WA, Robinson AS, Franz G (2011). Mitotic and polytene chromosomes analysis of the oriental fruit fly, *Bactrocera dorsalis* (Hendel) (Diptera: Tephritidae). Genetica.

[7] ASM78921v2 Genome Assembly [https://www.ncbi.nlm.nih.gov/assembly/GCF_000789215.1]. Accessed 11 February 2020.

[8] Handler AM, McCombs SD (2000). The *piggyBac* transposon mediates germ-line transformation in the oriental fruit fly and closely related elements exist in its genome. Insect Mol Biol.

[9] Sim SB, Kauwe AN, Ruano REY, Rendon P, Geib SM (2019). The ABCs of CRISPR in Tephritidae: developing methods for inducing heritable mutations in the genera Anastrepha, Bactrocera and Ceratitis. Insect Mol Biol.

[10] Bridges CB (1916). Non-disjunction as proof of the chromosome theory of heredity. Genetics.

[11] Carvalho AB, Koerich LB, Clark AG (2009). Origin and evolution of Y chromosomes: *Drosophila* tales. Trends Genet.

[12] Willhoeft U, Franz G (1996). Identification of the sex-determining region of the *Ceratitis capitata* Y chromosome by deletion mapping. Genetics.

[13] Meccariello A, Salvemini M, Primo P, Hall B, Koskinioti P, Dalíková M, Gravina A, Gucciardino MA, Forlenza F, Gregoriou ME (2019). Maleness-on-the-Y (MoY) orchestrates male sex determination in major agricultural fruit fly pests. Science.

[14] Buchman A, Akbari OS (2019). Site-specific transgenesis of the *Drosophila melanogaster* Y-chromosome using CRISPR/Cas9. Insect Mol Biol.

[15] Kaiser VB, Bachtrog D (2010). Evolution of sex chromosomes in insects. Annu Rev Genet.

[16] Rice WR (1996). Evolution of the Y sex in animals: Y chromosomes evolve through the degeneration of autosomes. Bioscience.

[17] Charlesworth B, Charlesworth D (2000). The degeneration of Y chromosomes. Philos Trans R Soc Lond Ser B Biol Sci.

[18] Bachtrog D (2013). Y-chromosome evolution: emerging insights into processes of Y-chromosome degeneration. Nat Rev Genet.

[19] Mahajan S, Bachtrog D (2017). Convergent evolution of Y chromosome gene content in flies. Nat Commun.

[20] Vibranovski MD, Koerich LB, Carvalho AB (2008). Two new Y-linked genes in *Drosophila melanogaster*. Genetics.

[21] Carvalho AB, Vicoso B, Russo CA, Swenor B, Clark AG (2015). Birth of a new gene on the Y chromosome of *Drosophila melanogaster*. Proc Natl Acad Sci U S A.

[22] Gabrieli P, Falaguerra A, Siciliano P, Gomulski LM, Scolari F, Zacharopoulou A, Franz G, Malacrida AR, Gasperi G. Sex and the single embryo: early development in the Mediterranean fruit fly, *Ceratitis capitata*. BMC Dev Biol. 2010;10:12. 10.1186/1471-213X-10-12 PMC2826288 20102629

[23] Champer J, Buchman A, Akbari OS (2016). Cheating evolution: engineering gene drives to manipulate the fate of wild populations. Nat Rev Genet.

[24] Sinkins SP, Gould F (2006). Gene drive systems for insect disease vectors. Nat Rev Genet.

[25] Hall AB, Qi Y, Timoshevskiy V, Sharakhova MV, Sharakhov IV, Tu Z (2013). Six novel Y chromosome genes in *Anopheles* mosquitoes discovered by independently sequencing males and females. BMC Genomics.

[26] Carvalho AB, Clark AG (2013). Efficient identification of Y chromosome sequences in the human and *Drosophila* genomes. Genome Res.

[27] Lisitsyn N, Wigler M (1993). Cloning the differences between two complex genomes. Science.

[28] Gabrieli P, Gomulski LM, Bonomi A, Siciliano P, Scolari F, Franz G, Jessup A, Malacrida AR, Gasperi G (2011). Interchromosomal duplications on the *Bactrocera oleae* Y chromosome imply a distinct evolutionary origin of the sex chromosomes compared to *Drosophila*. PLoS One.

[29] Torti C, Gomulski LM, Moralli D, Raimondi E, Robertson HM, Capy P, Gasperi G, Malacrida AR (2000). Evolution of different subfamilies of *mariner* elements within the medfly genome inferred from abundance and chromosomal distribution. Chromosoma.

[30] Torti C, Gomulski LM, Bonizzoni M, Murelli V, Moralli D, Guglielmino CR, Raimondi E, Crisafulli D, Capy P, Gasperi G (2005). *Cchobo*, a *hobo*-related sequence in *Ceratitis capitata*. Genetica.

[31] Tsoumani KT, Drosopoulou E, Bourtzis K, Gariou-Papalexiou A, Mavragani-Tsipidou P, Zacharopoulou A, Mathiopoulos KD (2015). *Achilles*, a new family of transcriptionally active retrotransposons from the olive fruit fly, with Y chromosome preferential distribution. PLoS One.

[32] Averbeck KT, Eickbush TH (2005). Monitoring the mode and tempo of concerted evolution in the *Drosophila melanogaster* rDNA locus. Genetics.

[33] Drosopoulou E, Nakou I, Síchová J, Kubíčková S, Marec F, Mavragani-Tsipidou P (2012). Sex chromosomes and associated rDNA form a heterochromatic network in the polytene nuclei of *Bactrocera oleae* (Diptera: Tephritidae). Genetica.

[34] Bedo DG, Webb GC (1989). Conservation of nucleolar structure in polytene tissues of *Ceratitis capitata* (Diptera: Tephritidae). Chromosoma.

[35] Goday C, Selivon D, Perondini AL, Greciano PG, Ruiz MF (2006). Cytological characterization of sex chromosomes and ribosomal DNA location in *Anastrepha* species (Diptera, Tephritidae). Cytogenet Genome Res.

[36] Marchi A, Pili E (1994). Ribosomal RNA genes in mosquitoes: localization by fluorescence *in situ* hybridization (FISH). Heredity (Edinb).

[37] Brianti MT, Ananina G, Recco-Pimentel SM, Klaczko LB (2009). Comparative analysis of the chromosomal positions of rDNA genes in species of the *tripunctata* radiation of *Drosophila*. Cytogenet Genome Res.

[38] Choo A, Nguyen TNM, Ward CM, Chen IY, Sved J, Shearman D, Gilchrist AS, Crisp P, Baxter SW (2019). Identification of Y-chromosome scaffolds of the Queensland fruit fly reveals a duplicated *gyf* gene paralogue common to many *Bactrocera* pest species. Insect Mol Biol.

[39] Kataoka N, Bachorik JL, Dreyfuss G (1999). Transportin-SR, a nuclear import receptor for SR proteins. J Cell Biol.

[40] Allemand E, Dokudovskaya S, Bordonné R, Tazi J (2002). A conserved *Drosophila* transportin-serine/arginine-rich (SR) protein permits nuclear import of *Drosophila* SR protein splicing factors and their antagonist repressor splicing factor 1. Mol Biol Cell.

[41] Lagos D, Ruiz MF, Sánchez L, Komitopoulou K (2005). Isolation and characterization of the *Bactrocera oleae* genes orthologous to the sex determining *Sex-lethal* and *doublesex* genes of *Drosophila melanogaster*. Gene.

[42] Ruscica V, Bawankar P, Peter D, Helms S, Igreja C, Izaurralde E (2019). Direct role for the *Drosophila* GIGYF protein in 4EHP-mediated mRNA repression. Nucleic Acids Res.

[43] Chang CH, Larracuente AM (2019). Heterochromatin-enriched assemblies reveal the sequence and organization of the *Drosophila melanogaster* Y chromosome. Genetics.

[44] Bachtrog D, Mahajan S, Bracewell R (2019). Massive gene amplification on a recently formed *Drosophila* Y chromosome. Nat Ecol Evol.

[45] Vicoso B, Bachtrog D (2015). Numerous transitions of sex chromosomes in Diptera. PLoS Biol.

[46] Stratikopoulos EE, Augustinos AA, Petalas YG, Vrahatis MN, Mintzas A, Mathiopoulos KD, Zacharopoulou A (2008). An integrated genetic and cytogenetic map for the Mediterranean fruit fly, *Ceratitis capitata*, based on microsatellite and morphological markers. Genetica.

[47] Saul SH (1982). Rearing methods for the medfly, *Ceratitis capitata*. Ann Entomol Soc Am.

[48] Baruffi L, Damiani G, Guglielmino CR, Bandi C, Malacrida AR, Gasperi G (1995). Polymorphism within and between populations of *Ceratitis capitata*: comparison between RAPD and multilocus enzyme electrophoresis data. Heredity (Edinb).

[49] Altschul SF, Gish W, Miller W, Myers EW, Lipman DJ (1990). Basic local alignment search tool. J Mol Biol.

[50] *Bactrocera dorsalis* [https://i5k.nal.usda.gov/content/bactrocera-dorsalis]. Accessed 11 February 2020.

[51] Pimpinelli S, Berloco M, Fanti L, Dimitri P, Bonaccorsi S, Marchetti E, Caizzi R, Caggese C, Gatti M (1995). Transposable elements are stable structural components of *Drosophila melanogaster* heterochromatin. Proc Natl Acad Sci U S A.

