# Supplementary material for: Transcribed sex-specific markers on the Y chromosome of the oriental fruit fly, Bactrocera dorsalis
Source: BMC Genet. 2020 Dec 18;21(Suppl 2):125. doi: 10.1186/s12863-020-00938-z (PMC7747380; doi:10.1186/s12863-020-00938-z)
Supplement: Supplementary file 1 — Additional file 1: Table S1. Primer pairs based on the 4 contigs. [file 12863_2020_938_MOESM1_ESM.docx]

**Additional file 1 - Table S1**. Primer pairs based on the 4 contigs.

| **Contig** | **Primer** | **Sequence (5' - 3')** | **T_a_ (°C)** | **Product (bp)** |
| --- | --- | --- | --- | --- |
| 1 original | contig1f | AAAGTTGCCAATGAAAAGTGTC | 56 | 120 |
|  | contig1r | TAGCTCGCAGAATATTATGTATGG | 56 |  |
|  |  |  |  |  |
| 2 original | contig2f | CTCAGAACCTGTTACGACAAAA | 57 | 125 |
|  | contig2r | TAAGCTGGGTTGTCATAGACG | 57 |  |
|  |  |  |  |  |
| 3 original | contig3f | AGTGGGCCTATCACAAACCA | 59 | 187 |
|  | contig3r | GATCACATCGCCTCGCTAGT | 59 |  |
|  |  |  |  |  |
| 4 original | contig4f | GGATGACGTGTCTGCCATC | 58 | 130 |
|  | contig4r | GCTGCGGATTAGCCTACTTG | 58 |  |
|  |  |  |  |  |
| 1 extended | contig1-171/358f | ACTTAAGAACACCTCGATCGAACA | 60 | 358 |
|  | contig1-358r | AGCTTTAACTTTAGTTTGCTACGTGC | 60 |  |
|  |  |  |  |  |
|  | contig1-171/358f | ACTTAAGAACACCTCGATCGAACA | 60 | 171 |
|  | contig1-171r | GGATTCTTTGCCGATGCGCACG | 65 |  |
|  |  |  |  |  |
| 2 extended | contig2-528f | GGCAGTGGAGTGGGCAGTACTG | 65 | 528 |
|  | contig2-528r | TGTTCAACCATTTGTCGACGCT | 61 |  |
|  |  |  |  |  |
|  | contig2-183f | ACTTTGGAGCAAGGCGATCTGC | 64 | 183 |
|  | contig2-183r | TCGACGTCGATCCGCTTCGT | 64 |  |
|  |  |  |  |  |
| 3 extended | contig3-701f | TGCCGAAATACAAGCAGAAGAAGCT | 63 | 701 |
|  | contig3-701r | TGGTCGCATTTTACACCGCCCA | 65 |  |
|  |  |  |  |  |
|  | contig3-156f | GCTTGGGGTTCAACAAACACAACTGT | 64 | 156 |
|  | contig3-156r | GGTTGCTTTACCGGGCATACGCA | 66 |  |
|  |  |  |  |  |
| 4 extended | contig4-1058f | GACCCACCTCCTTCACCACCTTG | 64 | 1058 |
|  | contig4-107/1058r | TGTCCTTGTGCGGGAAGTGCTAG | 64 |  |
|  |  |  |  |  |
|  | contig4-107f | CCCCCGAGTCAACCGATGTGAAA | 64 | 107 |
|  | contig4-107/1058r | TGTCCTTGTGCGGGAAGTGCTAG | 64 |  |
|  |  |  |  |  |
| NW_011875054.1 | NW_011875054.1f | ACTCACCAACACTGCCAACA | 61 | 180 |
| -specific | NW_011875054.1r | TCGACGTCGATCCTCTTCAT | 58 |  |
|  |  |  |  |  |
| Actin | Bdactinf | GGTCGGTATGGGACAGAAGG | 61 | 220 |
|  | Bdactinr | CTCACGATTGGCTTTTGGAT | 61 |  |
|  |  |  |  |  |
| MoY | BdMoYf | AAATGATATAGAAGAGCATGGG | 58 | 100 |
|  | BdMoYr | CGTTGAGAAGAGGTTAGTAT | 56 |  |
