# Supplementary figures and images for: Transcribed sex-specific markers on the Y chromosome of the oriental fruit fly, Bactrocera dorsalis
Source: BMC Genet. 2020 Dec 18;21(Suppl 2):125. doi: 10.1186/s12863-020-00938-z (PMC7747380; doi:10.1186/s12863-020-00938-z)

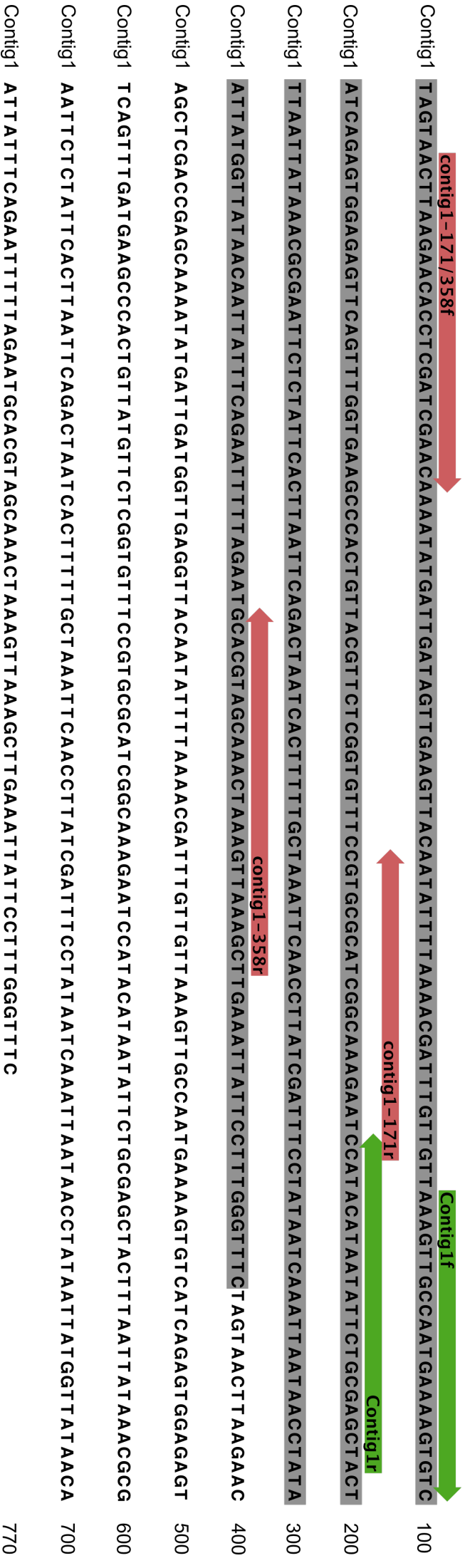

Supplement: Supplementary file 2 — Additional file 2: Figure S1. Sequence of the extended contig1 showing the positions of the primers. The grey box indicates the extent of the first repetition unit. [file 12863_2020_938_MOESM2_ESM.pdf]
