# Supplementary material for: Transcribed sex-specific markers on the Y chromosome of the oriental fruit fly, Bactrocera dorsalis
Source: BMC Genet. 2020 Dec 18;21(Suppl 2):125. doi: 10.1186/s12863-020-00938-z (PMC7747380; doi:10.1186/s12863-020-00938-z)
Supplement: Supplementary file 3 — Additional file 3: Figure S2. Sequence of the extended contig2 showing the positions exons (yellow rectangles) and primers. [file 12863_2020_938_MOESM3_ESM.pdf]

contig2-528f

Contig2 TAGGCAGTGGAGTGGGCAGTACTGAAGCTTGGCGCCGTAATATTGGCACAGGCTATAATTTCTCAGTAACACATCATTGGGGTATATAATAATAAA 96

G S G V G S T E A W R R N I G T G Y N F S V T H H W

contig2f

Contig2 TTCATTTTAAATTGCACGAAATATATTTGTCGTTATTTAATAGAACCACCTAATGAAAAATCCTCAGAACCTGTTACGACAAAACTTGTACCACAT 192

E P P N E K S S E P V T T K L V P H

contig2-183f contig2r

Contig2 CCGCAAAACCTAATTCCGGTATCTATGTGGGATTTAGACACTTTGGAGCAAGGCGATCTGCCAACAGTNACGTCTATGACAACCCAGCTTAAAGGT 288

P Q N L I P V S M W D L D T L E Q G D L P T V T S M T T Q L K G

Contig2 GAAACTGTCGCTAAACACCCTAATGGTAGTACAAAACGTGCTTTCAAGCAACAGATAGTAACCACGAAGAACCCAAAGGTGTCTGCTTGTAACAAA 384

E T V A K H P N G S T K R A F K Q Q I V T T K N P K V S A C N K

contig2-183r

Contig2 AAAAAAAAAAACGAAGCGGATCGACGTCGAGATCAAGCCGAAGATAAACATCGAGAGAAGGAGGAAAAGAAACGGCAGCAATCAAGTGAGGAAAAA 480

K K K N E A D R R R D Q A E D K H R E K E E K K R Q Q S S E E K

contig2-528r

Contig2 CGCCACCAACAACAATTAGATGATGAAAAGCGTCGACAAATGGTTGAACAAAATAAACGGCAGCTGCAAATTC 553

R H Q Q Q L D D E K R R Q M V E Q N K R Q L Q I
