# Supplementary material for: Transcribed sex-specific markers on the Y chromosome of the oriental fruit fly, Bactrocera dorsalis
Source: BMC Genet. 2020 Dec 18;21(Suppl 2):125. doi: 10.1186/s12863-020-00938-z (PMC7747380; doi:10.1186/s12863-020-00938-z)
Supplement: Supplementary file 4 — Additional file 4: Figure S3. Sequence of the extended contig3 showing the positions of an exon (yellow rectangle) and primers. [file 12863_2020_938_MOESM4_ESM.pdf]

```
        contig3-701f
Contig3 TAAAAGTTTTGCCGAAATACAAGCAGAAGAAGCTAAGTGTTTAGCCAATGAAAAAAATATGTTGAAGCAGCGTCGCAATAAACAGGAACAGCAGCA 96
         K  S  F  A  E  I  Q  A  E  E  A  K  C  L  A  N  E  K  N  M  L  K  Q  R  R  N  K  Q  E  Q  Q  H

                                                                                          contig3-156f
Contig3 CACGACAACTGTTACGAGTGCAACAAGTAATACGGGTGGAGGCCACAATAATATTTCGGCTGTATGTTCAAGCACAAAAGCTTGGGGTTCAACAAA 192
         T  T  T  V  T  S  A  T  S  N  T  G  G  G  H  N  N  I  S  A  V  C  S  S  T  K  A  W  G  S  T  N

        contig3-156f
Contig3 CACAACTGTTTTCTGGGACAAATCTATCAAATTTAGTGCTGTGGTTGCTGCTAATAATAATTCTGGTGGAAAGTAACTAAACAATCTTCAACCGGA 288
         T  T  V  F  W  D  K  S  I  K  F  S  A  V  V  A  A  N  N  N  S  G  G  K  *

                                   contig3-156r              Contig3f
Contig3 CAACAAAAACTTTCACCATCTGCGTATGCCCGGTAAAGCAACCATTGCAGTTAGTGGGCCTATCACAAACCAGCAGCTAAATACCGTACGTATAAT 384

Contig3 AGAGATAGATGTAGATAAATAATTGAGTAATGCACCGCGAYCTTAGGTCTATTGTGCCCTCTCCTAACTCACAGGGACTCACTTAGCCCCAGCATA 480

                                               Contig3r
Contig3 TTGATGAAGTCTAATATCCTGCTGGGTACTAGCGAGGCGATGTGATCCCTATCCGGAARCATGGATCCAAGGGCCTTCGACCAGCGCCTGCAGACT 576

Contig3 GCGATGCAGTCGACTAGCAGGTGCTCCGGGGTTTCAGGCTCCCTGTCGCAAAACCGACAGGTATCGCAATGATGATAGGCCCATGTTAGATAGATG 672

                              contig3-701r
Contig3 CCTTTTCAATTTGCAGTGGGCGGTGTAAAATGCGACCAGAAGCCTAAGTTTATCCCTTGGGAGGTT 738
```
