# Supplementary material for: Transcribed sex-specific markers on the Y chromosome of the oriental fruit fly, Bactrocera dorsalis
Source: BMC Genet. 2020 Dec 18;21(Suppl 2):125. doi: 10.1186/s12863-020-00938-z (PMC7747380; doi:10.1186/s12863-020-00938-z)
Supplement: Supplementary file 5 — Additional file 5: Figure S4. Sequence spanning contigs 2 and 3 (including the complete contig 2 and 3 sequences). [file 12863_2020_938_MOESM5_ESM.pdf]

Contig2-528f

contig2-contig3 T AGGCAGTGGAGT GGGCAGT ACTGAAGCT TGGCGCCGT AATAT TGGCACAGGCT ATAAT TT CTCAGT AACACATCAT TGGGGT ATAT AATAAT AAA 96

G S G V G S T E A W R R N I G T G Y N F S V T H H W

contig2f

contig2-contig3 T TCAT TT TAAAT TGCACGAAAT ATAT TTGT CGTT ATT TAAT AGAACCACCTAATGAAAAAT CCTCAGAACCTGT TACGACAAAACT TGT ACCACAT 192

E P P N E K S S E P V T T K L V P H

Contig2-183f contig2r

contig2-contig3 CCGCAAAACCT AATT CCGGTAT CTATGTGGGAT TT AGACACTT TGGAGCAAGGCGAT CTGCCAACAGTGACGT CTATGACAACCCAGCT TAAAGGT 288

P Q N L I P V S M W D L D T L E Q G D L P T V T S M T T Q L K G

contig2-contig3 GAAACTGT CGCTAAACACCCT AATGGT AGTACAAAACGTGCT TT CAAGCAACAGATAGT AACCACGAAGAACCCAAAGGTGT CTGCT TGT AACAAA 384

E T V A K H P N G S T K R A F K Q Q I V T T K N P K V S A C N K

Contig2-183r

contig2-contig3 AAAAAAAAAAACGAAGCGGAT CGACGTCGAGAT CAAGCCGAAGATAAACAT CGAGAGAAGGAGGAAAAGAAACGGCAGCAATCAAGTGAGGAAAAA 480

K K K N E A D R R R D Q A E D K H R E K E E K K R Q Q S S E E K

Contig2-528r

contig2-contig3 CGCCACCAACAACAAT TAGATGATGAAAAGCGT CGACAAATGGT TGAACAAAAT AAACGGCAGCTGCAAAT TCT AGTACGGCGGCGGCAAGCAAT T 576

R H Q Q Q L D D E K R R Q M V E Q N K R Q L Q I L V R R R Q A I

contig2-contig3 CT AGGAAATAAAGCCAATGT AAAAAAAT CTGGT TT AAGTGAAT CAGTGAAT TCGAGT TCT AATCT TCCT AACAAAAATCCGCAT TT CCAATCT TCT 672

L G N K A N V K N S G L S E S V N S S S N L P N K N P H F Q S S

contig2-contig3 GT TGCACCATGGT TT ACTCAAAGCAATGCAGCT TCT TCAGAAGGTGTGCCATGT CTAGCTGAAAT ACAAAAAGCTGAAAGACGGGAACGCCAAATG 768

V A P W F T Q S N A A S S E G V P C L A E I Q K A E R R E R Q M

contig2-contig3 GAACAGCGT AAAATGCGGCTGT TT CACAAACGAGTT CGTGCT AGTGCT AACGCTGCTGT TGAAGCAT TTGAT TCCT TGT TAAAATGGAACGT ACCT 864

E Q R K M R L F H K R V R A S A N A A V E A F D S L L K W N V P

Contig3-701f

contig2-contig3 GT AAGGGACGTCCCCGT TAAAAGT TT TGCCGAAAT ACAAGCAGAAGAAGCTAAGTGT TT AGCCAATGAAAAAAAT ATGT TGAAGCAGCGT CGCAAT 960

V R D V P V K S F A E I Q A E E A K C L A N E K N M L K Q R R N

contig2-contig3 AAACAGGAACAGCAGCACACGACAACTGT TACGAGTGCAACAAGT AATACGGGTGGAGGCCACAAT AATAT TT CGGCTGT ATGT TCAAGCACAAAA 1056

K Q E Q Q H T T T V T S A T S N T G G G H N N I S A V C S S T K

Contig3-156f

contig2-contig3 GCT TGGGGT TCAACAAACACAACTGT TT TCTGGGACAAAT CTAT CAAAT TTAGCGCTGTGGT TGCTGCT AATAAT AATT CTGGTGGAAAGT AACTA 1152

A W G S T N T T V F W D K S I K F S A V V A A N N N S G G K *

Contig3-156r contig3f

contig2-contig3 AACAAT CTT CAACCGGACAACAAAAACTT TCACCAT CTGCGT ATGCCCGGT AAAGCAACCATTGCAGT TAGTGGGCCT ATCACAAACCAGCAGCT A 1248

contig2-contig3 AAT ACCGTACGT ATAAT AGAGATAGATGT AGATAAAT AATTGAGT AATGCACCGCGAT CTT AGGTCT ATTGTGCCCT CTCCT AACTCACAGGGACT 1344

contig3r

contig2-contig3 CACT TAGCCCCAGCAT ATTGATGAAGT CTAAT ATCCTGCTGGGT ACTAGCGAGGCGATGTGAT CCCTAT CCGGAANCATGGAT CCAAGGGCCTT CG 1440

contig2-contig3 ACCAGCGCCTGCAGACTGCGATGCAGT CGACTAGCAGGTGCT CCGGGGTT TCAGGCT CCCTGT CGCAAAACCGACAGGTAT CGCAATGATGAT AGG 1536

Contig3-701r

contig2-contig3 CCCATGT TAGAT AGATGCCT TT TCAAT TTGCAGTGGGCGGTGT AAAATGCGACCAGAAGCCT AAGTT TAT CCCTTGGGAGGTT 1619
