# Supplementary material for: Transcribed sex-specific markers on the Y chromosome of the oriental fruit fly, Bactrocera dorsalis
Source: BMC Genet. 2020 Dec 18;21(Suppl 2):125. doi: 10.1186/s12863-020-00938-z (PMC7747380; doi:10.1186/s12863-020-00938-z)
Supplement: Supplementary file 6 — Additional file 6.Figure S5. Sequence of the extended contig4 showing the positions of a putative exon (yellow rectangle) and primers. [file 12863_2020_938_MOESM6_ESM.pdf]

```
                 contig4-1058f
Contig4  TAAGCGGCCGAACATCATGGACCCTCACTCCAAGTGAGGGACCCACCTCCTTCACCACCTTGTTGATAACCTCCTTAGAGGAAGTTTCAGGCCCCTTGCT  100
          L  P  R  V  D  H  V  R  V  G  L  S  P  G  V  E  K  V  V  K  N  I  V  E  K  S  S  T  E  P  G  K  S

Contig4  CCTCACAACCACCGACCAAGTCTCCACCGGCTTCGGCGTAACCGACGCAATAGCCCCCAGCACCTGTGCTGCATGCGCATGCATCGGTGCCGACGGAACC  200
          R  V  V  V  S  W  T  E  V  P  K  P  T  V  S  A  I  A  G  L  V  Q  A  A  H  A  H  M  P  A  S  P  V

Contig4  GGCAAAGACCCACCCGGCGGCGAAAAGTGCCCCCCGCAACTTCCCCTAAGGGCTTCTACCTGACCGCGAAGATGGGCATTCTCGGCTATAACCGTCATCA  300
         P  L  S  G  G  P  P  S  F  H  G  G  C  S  G  R  L  A  E  V  Q  G  R  L  H  A  N  E  A  I  V  T  M  L

Contig4  AGAGCGCCTCATATTTTGAGGCAAACTCCAGCAGCCCCTTCGCGACCTCTTTCTCAAAATTTACCGCCCCAAATACCAACTCACACAGCTCAACATTCAA  400
          L  A  E  Y  K  S  A  F  E  L  L  G  K  A  V  E  K  E  F  N  V  A  G  F  V  L  E  C  L  E  V  N  L

Contig4  TTTTTCGCAGCCACAACATGGCTTGTTGCCCCCTACCCTTACTCCCAACACTACCGTCACGCTTGCCGCCTCACTTGTTCAGTGGTGCCACCCTTACCA  500
          K  K  A  A  V  V  H  S

Contig4  CCCCCACTACCTCTACCACTCCCGCTCCATCCCCCATCGCTACGCTCCGCACCTGGAGACGCCAAGCGCGCTTTGCGCTTCGGTGATGACCTCAACGGAG  600

                      contig4f
Contig4  GTCTCATCCCCTCATCCCCGGATGACGTGTCTGCCATCACGGTCGCAGGCCCTGACCCATCACTCTCAGCGAGGGAGTCAAACCTCTTTTTCCGTGGCAG  700

                                           contig4r
Contig4  AGGCATCCCCACCTTCTGAACTTACCCACCAAGTAGGCTAATCCGCAGCAAACAAAAAACCACTAACTCAGTACCACGAGTACAAAGGAAAAACCCGGAA  800

Contig4  CAGCTGCTCGCAAGGCGTGTGACTGAACACACAGAAAAAGCTATCAAGAACGCAAGAATGCCCAGTAGCAGTCGCCACCCAAGCGGTCGACTAAGGCGAA  900

                                                                                                   contig4-107f
Contig4  AAAACGCGAGGGCACACCCCACAACCCAAGCAGAAGCCCGGAAAGGCCACTCAAAAACGGGTGCCGGACGGAACTTTGTCCCCGTACCACCCCCGAGTCA  1000

         contig4-107f                                                                  contig4-107/1058r
Contig4  ACCGATGTGAAAAATGCGCCGAGTCACACCGTGGGCTCAAAAGCAGACCACTACGACCAAACAAGTCGCAAAGCTAGCACTTCCCGCACAAGGACAGGCA  1100

Contig4  CCAGCACCTTT                                                                                           1111
```
