# Supplementary material for: Transcribed sex-specific markers on the Y chromosome of the oriental fruit fly, Bactrocera dorsalis
Source: BMC Genet. 2020 Dec 18;21(Suppl 2):125. doi: 10.1186/s12863-020-00938-z (PMC7747380; doi:10.1186/s12863-020-00938-z)
Supplement: Supplementary file 7 — Additional file 7: Figure S6. Alignment of part of the extended contig2 and Scaffold01347 (NW_011875054.1), indicating the positions of primers designed to amplify preferentially the NW_011875054.1 sequence. [file 12863_2020_938_MOESM7_ESM.pdf]

```
              NW_011875054f →
NW_011875054  GAAGAACTCACCAACACTGCCAACAGCATCATCTCTGACAAAATTGCCAAAAGATGAAACTGTTGGTAAACACCATAATA   80
Contig2       TTTGGAGCAAGGCGATCTGCCAACAGTNACGTCTATGACAACCCAGCTTAAAGGTGAAACTGTCGCTAAACACCCTAATG   80

NW_011875054  TTAGTACAAAACGTGCGATCAAGCAACAGCTAGTAACCGGTAAGAACTCAAAGGTGTCTGAGGACAGTGCTTGCAGC---  157
Contig2       GTAGTACAAAACGTGCTTTCAAGCAACAGATAGTAACCACGAAGAACCCAAAGGTGTC---------TGCTTGTAACAAA  151

                    ← NW_011875054r
NW_011875054  ---AAAAAAAATGAAGAGGATCGACGTCGAGATCAAGCTGAAGATAAGCGTCGGCAGAAGGAGGAAAAGAAACGCCAGCA  234
Contig2       AAAAAAAAAAACGAAGCGGATCGACGTCGAGATCAAGCCGAAGATAAACATCGAGAGAAGGAGGAAAAGAAACGGCAGCA  231

NW_011875054  AGCAGATGAGGAAAAACGCCGCCAACAACAATTAGATGATGAAAAGCGTCGACAAATGCTTGAAGAAAAAGAACGGCAGC  314
Contig2       ATCAAGTGAGGAAAAACGCCACCAACAACAATTAGATGATGAAAAGCGTCGACAAATGGTTGAACAAAATAAACGGCAGC  311
```
