# Supplementary material for: Transcribed sex-specific markers on the Y chromosome of the oriental fruit fly, Bactrocera dorsalis
Source: BMC Genet. 2020 Dec 18;21(Suppl 2):125. doi: 10.1186/s12863-020-00938-z (PMC7747380; doi:10.1186/s12863-020-00938-z)
Supplement: Supplementary file 8 — Additional file 8: Table S2. Primers for Representational Difference Analysis (RDA). The R- primers were used in the preparation of the initial amplicon representations, and the J- and N- primers were used for odd and even hybridization-amplifications, respectively. [file 12863_2020_938_MOESM8_ESM.docx]

**Additional file 8 - Table S2**. Primers for representational difference analysis (RDA). The R- primers were used in the preparation of the initial amplicon representations, and the J- and N- primers were used for odd and even hybridization-amplifications, respectively.

| **Primer** | **Sequence (5' - 3')** |
| --- | --- |
| R-Msp24 | AGCACTCTCCAGCCTCTCACCGCAC |
| R-Msp12 | CGGTGCGGTGAG |
| J-Msp24 | ACCGACGTCGACTATCCATGAACAC |
| J-Msp12 | CGGTGTTCATGG |
| N-Msp24 | AGGCAACTGTGCTACTCGAGGGAAC |
| N-Msp12 | CGGTTCCCTCGA |
| R-Mse24 | AGCACTCTCCAGCCTCTCACCGCAT |
| R-Mse12 | TAATGCGGTGAG |
| J-Mse24 | ACCGACGTCGACTATCCATGAACAT |
| J-Mse12 | TAATGTTCATGG |
| N-Mse24 | AGGCAACTGTGCTACTCGAGGGAAT |
| N-Mse12 | TAATTCCCTCGA |
