# Supplementary material for: Transcribed sex-specific markers on the Y chromosome of the oriental fruit fly, Bactrocera dorsalis
Source: BMC Genet. 2020 Dec 18;21(Suppl 2):125. doi: 10.1186/s12863-020-00938-z (PMC7747380; doi:10.1186/s12863-020-00938-z)

Figure 1 A

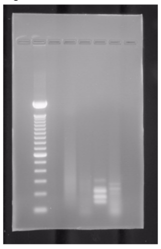

Figure 1 B

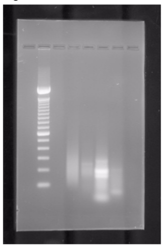

Figure 2 Contig 1

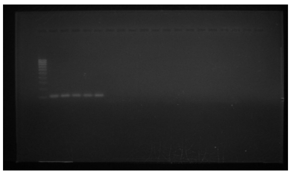

Figure 2 Contig 2

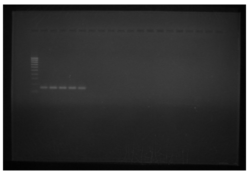

Figure 2 Contig 3

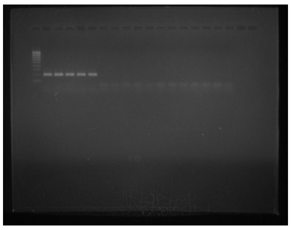

Figure 2 Contig 4

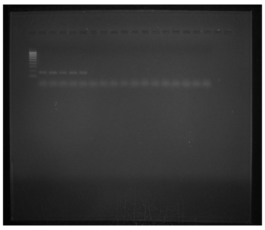

Figure 2 Actin

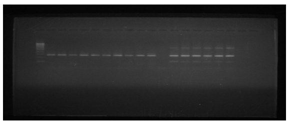

Figure 3 A Contig 1

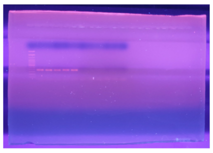

Figure 3 A Contig 2

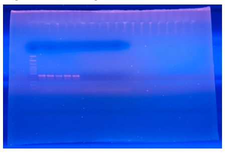

Figure 3 A Contig 3

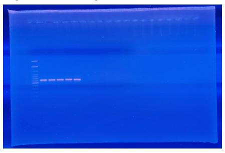

Figure 3 A Contig 4

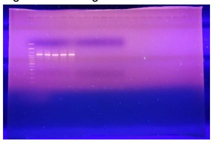

Figure 3 A NW_011875054.1 specific

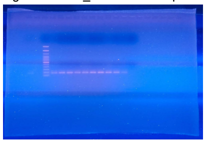

Figure 3 A Actin

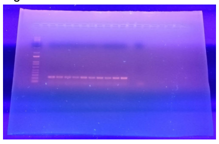

Figure 3 B Contig 1

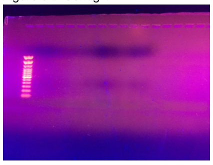

Figure 3 B Contig 2 (top) and Contig 4

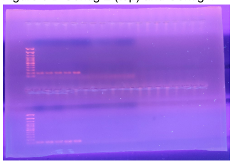

Figure 3 B Contig 3

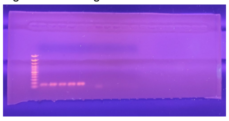

Figure 3 B NW_011875054.1 specific

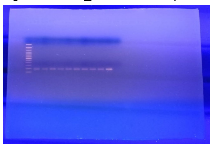

Figure 3 B Actin

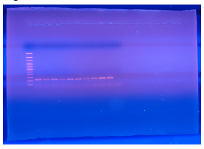

Figure 4 B Contig2f + Contig3r

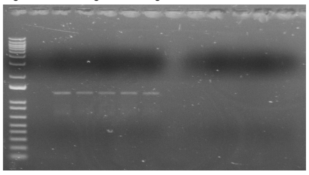

Figure 7 Contig 1 24-48h embryos

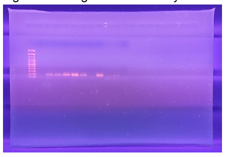

Figure 7 MoY 24-48h embryos

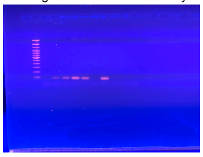

Figure 7 Actin 24-48h embryos

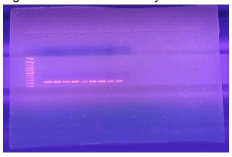

Figure 7 Adults Contig 1, MOY and Actin

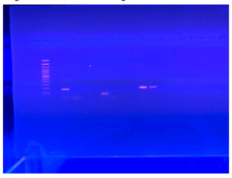

Supplement: Supplementary file 9 — Additional file 9. Unedited gel images from Figs. 1, 2, 3, 4 and 7. [file 12863_2020_938_MOESM9_ESM.pdf]
